# A review of official data obtained from dog control records generated by the dog control service of county cork, Ireland during 2007

**DOI:** 10.1186/2046-0481-65-10

**Published:** 2012-06-08

**Authors:** Edmond N O’Sullivan, Alison J Hanlon

**Affiliations:** 1 Veterinary Department, Cork County Council, County Hall, Cork, Ireland; 2 School of Veterinary Medicine, University College Dublin, Belfield, Dublin 4, Ireland

**Keywords:** Stray dogs, Official control records, Breed population, Aggression, Responsible

## Abstract

**Background:**

There are no peer reviewed data on dog control records from an official agency in Ireland. In order to address this, a total of 2,669 official dog control service records generated during 2007 by Cork County Council dog control service were reviewed.

**Results:**

Over 70 percent of records related to unwanted dogs and dogs not under their owners control. Stray dogs were collected by the service regularly throughout the year but with notable increase in voluntary surrenders by owners from January through to April. The majority of dogs collected or surrendered were male (2:1 ratio), of medium size, described as having a friendly temperament and were not wearing a neck collar. The Crossbreed and Greyhound breeds were more frequently collected as strays, while Greyhounds and German Shepherds were more frequently voluntarily surrendered by their owner. Restricted breeds such as Pit Bull terriers, German Shepherds and Rottweilers were more frequently reported by members of the public for aggressive behaviour while the only restricted breed reported for biting or snapping was the German Shepherd.

**Conclusions:**

Routine recording of dog control services in County Cork provide data on responsible dog ownership including the licensing of breeds, and surrender of owned dogs and the collection of stray dogs. Data capture and utilisation of dog control services by local authorities has potential to inform policy on responsible dog ownership and education programmes.

## Background

The world population of domestic dogs is approximately 500 million and it is considered that a substantial number of this population of dogs are stray, owned but free-roaming or inadequately supervised [1,2]. In 2011 in the UK, dog control authorities handled 1 stray dog for every 465 persons in the national population [3].

Legislation specific to dogs is introduced for a variety of reasons. Free roaming dogs have long caused significant public health and animal welfare concerns in many countries [4]. According to the International Companion Animal Management Coalition [5], members of the public and government authorities are concerned about public health and safety issues associated with straying dogs. These issues include transmission of disease to humans and other animals, injury and fear caused by aggressive behaviour, nuisance due to noise and fouling, accidents and livestock worry. Some 30–40 diseases of companion animals are transmissible to humans [6]. Consequently, responsible pet ownership has become an increasing concern of medical professionals and national, state and community officials [7].

Factors which contribute to the stray and/or free roaming dog population include low neutering rates, ready access to low or no-cost puppies, canine behavioural problems, unrealistic expectations of dog ownership and irresponsible dog ownership [1,2,8].

In 2008, the World Health Organisation (WHO) commissioned a report on Stray Animal Control Practices in 31 European and Eurasian countries [9]. The report found that 13 countries had national legislation that specifically addressed pet ownership, 24 had legislation relating to stray animals and 22 countries had legislation relating to dangerous or aggressive dogs. The same report concluded that those countries with no or low numbers of stray dogs had legislation that is effectively enforced. In a review of data obtained from 81 OIE member countries, Dalla Villa et al. reported that dog registration/licensing was the most frequent method used for dog control, and that in 72% of OIE countries, local authorities were responsible for implementing dog control [10]. In addition, these reports commented that there is a universal lack of stray animal control data collected by authorities [9] and an absence of published studies on dog control data [10].

In the UK, there is an attempt to collect stray dog data from local authority dog control services by means of an annual voluntary survey carried out by the dog charity “Dogs Trust” [3]. This takes the form of an e-mail or postal questionnaire sent to all 404 local authorities in the UK. In Ireland, stray dog collection, surrender, licensing, re-homing, euthanasia and enforcement (fines) data collected from 34 local authority dog control services is published annually by the Central competent authority [11].

In Ireland in 1986, as a response to a number of high profile livestock kills, the Control of Dogs Act was introduced. This act is implemented by local authorities and requires that all dogs must be licensed and kept under effectual control. County Cork is the largest local authority area in Ireland and the county Dog Control Act functions are carried out and administered by the Veterinary Department. In 2007, there were 32,550 individual and 97 group (general) dog licenses issued, respectively, in County Cork [11]. Dog ownership in Ireland is relatively high. A recent study [12] found that 35.6% of Irish households owned one or more dogs compared to 21% in the UK [13]. In Ireland, rural households, particularly small farmers, and those with children of school age are more likely to own dogs [12]. The same authors contend that there is a relatively large stray dog population in Ireland but suggest that further data on the stray dog population is required.

There are no peer reviewed data relating to dog control in Ireland. The objective of this paper is to review official dog control data generated by the Cork County Council dog control service during 2007 and discuss the value of these data to inform policy on responsible dog ownership.

## Materials and methods

Daily dog control duties in Cork County are carried out by one full time dog warden, eleven part-time wardens, two litter wardens with limited dog warden powers, two dog-pound keepers and three full-time administrative staff.

The service operates on the basis of a compulsory annual paper license required for all dogs over four months of age, policing of license compliance and responding to dog control issues reported by members of the public. Every service request is logged manually in the dog control day-book and the call is then relayed to the dog warden supervising the area concerned, to take appropriate action. The majority of these actions comprise the collection of stray dogs, the collection of voluntarily surrendered dogs, seizure of out of control dogs and responding to complaints regarding dog fouling and dog aggression. Routine reports are generated for these actions. In addition, dog wardens submit non-routine reports relating to particular service requests. All reports are archived by administrative staff for the purposes of internal audit and possible legal investigations.

All paper records generated by the dog control service from January 1^st^ 2007 to December 31^st^ 2007 were collated. The records were pre-screened for document completeness and a total of 123 documents were excluded. The records utilised comprised the dog control diary, non-routine incident reports and routine stray dog collection forms (Additional File 1) and dog surrender forms (Additional File 2).

All data was recorded in a Microsoft Excel database. Descriptive statistics were generated. In order to calculate breed specific population data, the county dog license database was utilised to establish individual breed populations during an equivalent 12 month period, 2005. It was not possible to obtain this data for 2007 due to recent alterations in the license data input programme. A total of 32,172 individual dog licenses were accessed on the county dog license database for 2005. Individual breed populations were calculated using the breed data provided on each license by the license applicant.

## Results

A total of 674 stray dog collection forms, 118 dog surrender forms and 2,103 service request entries were admitted to the study. Following assessment, the 2,103 service request entries resulted in a total of 2,669 categorisations. This increase is because a number of individual service request entries represented multiple categories.

The frequency of each service request category is given in Table 1.

**Table 1:** Categorisation of 2,669 service requests received by the dog control service, Cork County Council in 2007

**Service request category**	**Number**	**Percentage**
Collect a stray dog from a persons’ property	836	31.3
Owned dog out of control in public place	774	28.9
Bite incident/report of aggressive behaviour	361	13.5
Dog straying in a public place	248	9.2
Nuisance due to fouling	154	5.8
Owner voluntarily surrendering a dog	101	3.8
Concern about dog welfare	71	2.7
Livestock worry	62	2.3
Nuisance due to barking	45	1.7
Reporting a missing dog	17	0.6

A total of 75.5 per cent of the service requests related to dogs that were unwanted and/or out of the owners’ control. Of the 792 collection/surrender forms, 85% were for stray dog collection and 15% were for dog surrender. Two thirds of the total forms related to rural collection/surrender and one third were for dogs collected/surrendered in either an urban or sub-urban environment. One third (n = 258; 32.6%) of the 792 collection/surrender forms included additional information provided by the dog warden as to why the dog was admitted to the dog control service. In almost two thirds of cases, this was because the dog was reported to be loose on a public road (Table 2).

**Table 2:** Reason for the collection and surrender of dogs to the dog control service Cork County Council in 2007 (n = 258)

**Reason**	**Number**	**Percentage**
Loose on public road	167	64.7
Strayed onto business premises	20	7.7
Aggressive behaviour	19	7.4
Concern for its welfare	18	7
Strayed into a premises	13	5
Police request	8	3.1
Dog had bitten a person	7	2.7
Chasing cars/bicycles	6	2.3

**Figure 1:**
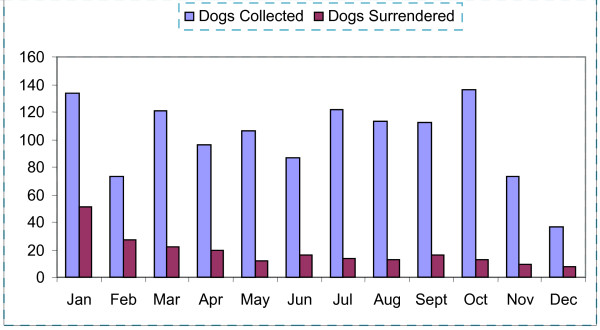
Monthly distribution of stray dog collection and owned dog surrenders to the dog control service in County Cork in 2007.

Time of year appeared to influence the frequency of dogs surrendered, with the highest number recorded in January. Surrender requests decreased from February to April and remained constant thereafter (Figure 1). Stray dog collections, both for adult dogs and dogs < 6 months of age, were generally constant throughout the year with the exception of a marked drop in December.

**Figure 2:**
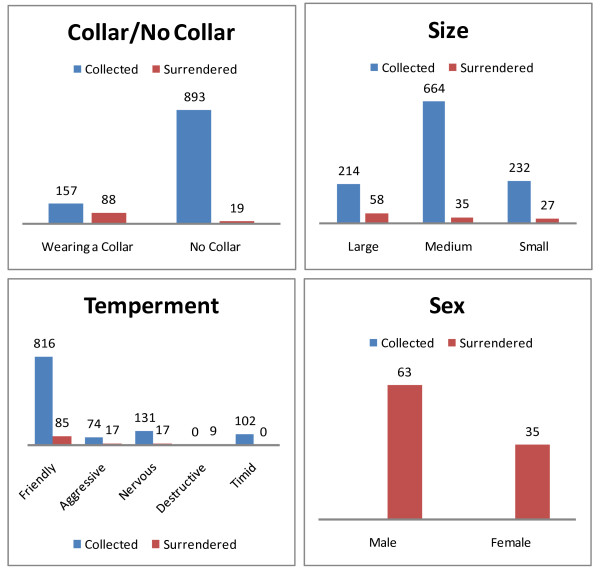
Information and assessment provided by dog wardens for stray and surrendered dogs entering the dog control service in County Cork in 2007.

The majority of dogs collected or surrendered were male (2:1 ratio), of medium size, of friendly temperament as assessed by the dog warden and were not wearing a neck collar (Figure 2).

**Figure 3:**
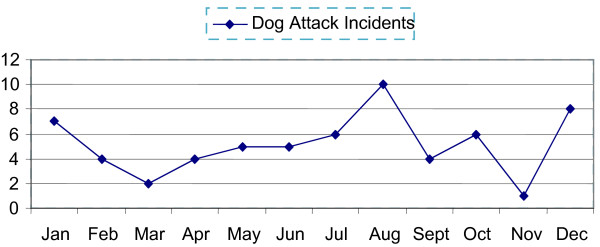
Monthly distribution of dog attacks on other animals reported to the dog control service in County Cork in 2007.

Reported incidents of dog attacks on other animals (livestock worry), included attacks on other dogs and cats as well as farm animals. While the highest frequency of attack reported was during August, no seasonal pattern was evident (Figure 3).

Utilising individual breed population data obtained from the county dog license database, Table 3 depicts the 6 most popular licensed breeds in County Cork with Collie type dogs accounting for almost a quarter of all licensed dogs.

**Table 3:** Six most popular dog breeds registered for a dog licence in County Cork in 2005

**Breed**	**No. licensed**	**% of total licensed**
Collie	7730	24
Terrier	4665	14.5
Labrador	3405	10.6
Jack Russell Terrier	2587	8
Cocker/Springer Spaniel	2222	7
Crossbreed	2068	6.4

The six most frequently collected and surrendered breeds are presented in Table 4 in conjunction with an estimated breed representation, determined by the dog licence database.

**Table 4:** The six breeds of dogs most frequently collected by dog wardens and their surrendered-by-their-owner figure expressed as a percentage of the total no. of that breed licensed in County Cork

**Breed**	**No. (%) collected**	**No. licensed**	**No. (%) surrendered**
Crossbreed	451 (22)	2068	0 (0)
Collie	223 (3)	7730	26 (0.3)
Terrier	132 (3)	4665	11 (0.2)
Labrador	97 (3)	3405	7 (0.2)
Greyhound	57 (4.5)	1200	48 (4)
German shepherd	30 (2)	1444	13 (1)

Table 5 details the six breeds of dogs most frequently reported to the dog control service because of incidents of aggressive behaviour while Table 6 shows the breeds reported for actually biting or snapping at a member of the public.

**Table 5:** The six breeds of dogs most frequently reported by the public for aggressive behaviour (n = 191) as obtained from the dog control day-book in County Cork in 2007

**Breed**	**No. licensed**	**No. reported**	**Breed aggression rate (%)**
German Shepherd	1444	75	5
Pit Bull Terrier	1	45	N/A
Rottweiler	365	30	8
Staffordshire Bull Terrier	106	16	15
Terrier	4665	13	0.2
Labrador	3405	12	0.3

**Table 6:** The six breeds of dogs most frequently reported by the public for biting or snapping (n = 119) as obtained from the dog control day-book in County Cork in 2007

**Breed**	**No. licensed**	**No. reported**	**Breed bite/snap rate (%)**
Unidentified	N/A	75	N/A
Terrier	4665	13	0.3
German Shepherd	1444	12	0.8
Labrador	3405	8	0.2
Retriever	1594	5	0.3
Springer	2222	3	0.1
Collie	7730	3	0.03

While the German Shepherd was the breed most frequently reported for aggressive behaviour incidents, when compared to the breed population recorded in the dog licence database, the Staffordshire Bull Terrier and the Rottweiler had a higher proportion of aggressive incidents than German Shepherds.

In over 60% of reports for biting/snapping, the person making the report was unable to describe the breed of dog involved. Where a specific breed was identified, the German Shepherd, a restricted/listed breed, was most frequently identified. Using breed population data, this breed had the highest incidence of biting and/or snapping.

## Discussion

Members of the public are motivated to contact an official agency for a variety of reasons. It is the authors’ experience that these can range from genuine concern regarding an issue through to disputes with dog owners. In addition, when interacting with an official agency, members of the public may place particular bias on, withhold or alter the information they provide.

Such bias may be evident in breed-recognition and is acknowledged as a limitation of this study. Breed identification obtained from the dog license database utilised in this study is reliant upon the owners’ knowledge of their dogs’ breed. In addition, dog license compliance is estimated to be 60% of the county dog population and thus some breeds may be more likely to be licensed, creating a further limitation to the study.

Despite these limitations, a descriptive data review of this nature may generate information on dog control issues that could be utilised to inform dog control strategies at local authority and national level and to complement existing responsible dog ownership education programmes. Key issues of responsible dog ownership include acquiring a breed appropriate to the household, understanding the behavioural needs of a dog, the husbandry and veterinary costs of owning a dog and being aware of the civic duties associated with dog ownership, e.g. nuisance barking and dog fouling.

The breed population data in the present study indicates that Crossbreeds, Collies, and Terriers constitute almost half of the licensed breeds in County Cork and these were the breeds most frequently presented to the dog control service. A US study of 2,631 dogs surrendered to rescue shelters found that crossbreeds were over-represented [14]. The dog wardens in Cork ordinarily describe Crossbreeds, Collies and Terriers as “farm-type dogs”. This may reflect both the large rural population of Irelands’ largest county and a recent significant demographic shift from rural to urban dwelling in Ireland [15]. It may be that former rural dwellers seek to retain these breeds as a connection with rural living [2].

An anomaly is apparent with the Pit Bull Terrier breed population data and reports of bites by this breed. This may be explained by an adverse public perception of Pit Bull Terriers. Owners of this breed may be unwilling to enter the breed type on the license and members of the public may wilfully or erroneously describe all bull terrier type dogs as being Pit Bull Terriers. Therefore the data quoted for Pit Bull Terriers and Staffordshire Bull Terriers may not be reliable. The limitations of this study mean that the significance of the data, particularly in regard to the German Shepherd breed apparent propensity for aggression, biting and snapping behaviour is difficult to assess.

In the present study, almost three quarters of official dog control duties related to dogs that were unwanted and/or not under the control of the owner. In a 2011 UK survey of stray dogs collected by local authorities, it was estimated that almost half of the stray dogs were re-united with their owners [3], while in the US it is estimated that one third of dogs entering shelters are owned dogs [8]. In their study of dog keeping in Taiwan, Hsu et al. [2] identified low rates of neutering, a ready supply of low or no cost puppies, a tendency to allow dogs free access to the outdoors and unrealistic expectations of dog ownership as having contributed to the problem of free-roaming dogs. The same authors identified “too much trouble to look after” and “behaviour problems” as the two most common reasons for abandoning a dog and concluded that many of the dog owners lacked basic understanding of dog behaviour and had unrealistic expectations of the time, effort and space needed by dogs. In a US study of owner’s reasons for surrendering dogs to shelters, moving house, landlord issues, costs and lack of time were the top four reasons [8]. Similar factors may apply in Ireland. For example, Downes et al., [12] reported a pet dog neutering rate of only 47.3%. Irish newspapers, particularly those with free advertising sections, carry a large number of advertisements for puppy sales. Interestingly, Hsu et al., [2] concluded that a recent transition from rural to pre-dominantly urban society in Taiwan created a situation where relatively detached rural attitudes towards dogs have been trans-located to an urban setting in which they are inappropriate. As stated above, a similar demographic shift has taken place in Ireland in recent years.

In the present study, while the collection of stray dogs was generally constant throughout the year, it is notable that voluntary surrenders peaked in January and tended to remain high through to April. While there would be a reduction in surrenders/collections during the immediate Christmas/New Year period due to dog warden holidays, it is the authors’ view that the January peak may reflect the inappropriate acquisition of dogs in the run-up to Christmas and/or the reassessment of household budgets after Christmas. This data confirms a long-established trend high-lighted by dog welfare charities.

Male dogs were over-represented in the dog control data. This may simply reflect the numerical predominance of male dogs in the county dog population (2:1). However, it may also represent either owner selection of male dogs because of aggression potential or more frequent behavioural problems and testosterone related issues associated with male dogs [16,17].

The reason for dog collection/surrenders identified in the present study resembles that of a 2011 study [3] of UK stray dog collection/surrenders. However, the latter describes stray dogs as “brought in by members of the public”, which may not amount to “surrender” of their own dog in all cases.

Crossbreeds and Collie dogs comprised the majority of dogs collected by the Cork County dog control service. It is interesting to note that the breed collection rate for Crossbreeds was almost one quarter, compared to 3% in Collies. This indicates that a large number of crossbreeds were unwanted by their owners and/or were not licensed. The proportion of crossbreeds collected may also reflect the acquisition pattern for dogs in Ireland. In Taiwan for instance, just over half of the dogs in one study had been acquired from friends and relatives and one fifth from rescue centres [2]. The relatively high number of Greyhounds in the collection and surrender categories probably reflects the commercial nature of Greyhound ownership. Once a Greyhound is no longer able to race satisfactorily, the owner, in view of the cost of euthanasia and the time and effort involved with re-homing, may resort to the less costly option of surrendering the dog to the dog control service.

Dog bite incident reports and fear of aggressive behaviour was the third most frequent service request in the present study. These reports related to bite/aggressive episodes in public places. A study in the Netherlands [18] concluded that almost 90 per cent of bite incidents in public places involved non-owners and most of the victims believed that the dog bit intentionally and was unprovoked. Similar findings were reported in a previous Irish study [19]. In recent years extensive media reportage of dog attacks has contributed to increased public fear of dogs. Attacks carried out by some of the restricted breeds (Additional File 3) or so called “dangerous dogs” may be attributed to public awareness of aggressive behaviour by these breeds. In the present study, this is supported by the difference in breeds reported for aggressive behaviour, mainly the restricted breeds, and the breeds reported for biting members of the public, the non-restricted breeds. Four (Pit Bull Terrier, Staffordshire Bull terrier, German Shepherd and Rottweiler) of the six breeds most frequently reported for aggressive behaviour comprised breeds that are on the restricted list in Ireland while one (German Shepherd) of the restricted breeds is to be found in the six breeds most frequently reported for actually biting or snapping at a person. Whilst restricted breeds may be more aggressive, this finding may also indicate that the experience of a growl or snarl from a restricted breed is more likely to result in a complaint to the authorities than would similar behaviour from a non-restricted breed. It is the authors’ view that this reflects the media derived perception amongst the public that the restricted breeds pose a more significant public danger. The over-representation of the German Shepherd breed, a restricted breed, in terms of breed bite/snap rate in the present study has been noted in other studies [20,21]. The latter authors ascribe this to a possible familial inclination to bite because of fear. More recently, an Irish study [19] of 234 bite incidents did not identify the German Shepherd in the six highest breed bite rate while in Holland, Cornelissen and Hopster [18] identified the breed as having the third highest bite rate index after the Rottweiler and Doberman breeds, who were first and second, respectively.

In addition, in the present study, the German Shepherd breed was second only to the Greyhound in terms of being voluntarily surrendered by the owner, thus perhaps indicating owner concern or difficulty with the breed. Given the popularity of this breed as a domestic pet as evidenced by the Cork County dog license database, these findings merit further investigation.

In the present study, complaints relating to dog fouling comprised the fifth most frequent service request. Dog fouling in public places is a significant dog control and public health concern [22,23] and in Ireland failure to remove and dispose of foul is in contravention of the 1997 Litter Pollution Act.

Noise nuisance due to dogs barking is generally perceived to be widespread. However, official reporting of this nuisance was low in the present study. Dog wardens in County Cork report that those most affected by this nuisance tend to be close neighbours of the dog owner and thus may be less likely to complain to an official authority. In addition it is likely that, in order to maintain a harmonious relationship with others in their community, responsible dog owners, on receipt of a complaint from a neighbour, would take appropriate measures to rectify the problem before it is reported to the dog control service authorities.

Livestock worry, as stated in the introduction, was the original reason for the introduction of dog control legislation in Ireland. The low number of service requests in this category suggests that legislation in this regard has been successful. The peak incidence in August is to be expected as this is the principle holiday time of year associated with, for example, children playing outside more with dogs, rental or use of holiday homes in rural areas by dog owners and members of the public walking their dogs in rural areas more frequently.

The current study demonstrates that key aspects of responsible dog ownership are routinely recorded by Cork County Council. An annual review of such data could be used to inform policy such as school education programmes on responsible dog ownership. While the present study did not formally investigate data capture and utilisation methods by other Local Authority dog control services in Ireland, one of the authors is aware that the methods used vary considerably. Standardisation of data capture and utilisation of dog control services would provide an opportunity to develop cohesive national policy and an improved approach to responsible dog ownership in Ireland.

## Competing interests

We declare that we have no competing interests.

## Authors’ contributions

EOS designed the study, acquired the data and carried out the analysis and interpretation of the data. AH carried out critical revision of each draft of the paper. All authors read and approved the final manuscript.

## Authors’ information

EOS has been working with stray dog control legislation since 1992. He was a past chairman of the Irish National Stray Dog Forum and is a current member of the Local Authority Dog Breeding Establishments Legislation advisory group.

## Supplementary Material

Additional File 1 Stray Dog form completed by dog wardens when collecting a stray dog. Click here for file

Additional File 2 Owned dog voluntary surrender form completed by the dog warden when collecting a dog from its owner. Click here for file

Additional File 3 List of the “Restricted breeds”. Click here for file
