# Supplementary material for: A review of official data obtained from dog control records generated by the dog control service of county cork, Ireland during 2007
Source: Ir Vet J. 2012 Jun 8;65(1):10. doi: 10.1186/2046-0481-65-10 (PMC3489852; doi:10.1186/2046-0481-65-10)
Supplement: Additional File 1 — Stray Dog form completed by dog wardens when collecting a stray dog. [file 2046-0481-65-10-S1.doc]

**Additional File 1.** Stray Dog form completed by dog wardens when collecting a stray dog

**Cork County Council**

**Control of Dogs Act, 1986/1992**

**Notification of Finding of Stray Dog**

Section A

Name of Finder of Dog…………..……………………………………………………………

Address…………………………………………………………………………………………

Contact no (optional)………………………………………………………………................

**Breed (if known)**…………………………………………..**Colour**………………..………..

Size of Dog: Large □ Medium □ Small □

Wearing a collar: Yes □ No □

Personality: Friendly □ Nervous □

Timid □ Aggressive □

Place where found …………………………………………………………………………...

Date of Notification …………..……………………………………………………………….

Date of seizure …………………….………………………………………………………….

Where kennelled ……………………….……………………………………………………..

Date placed in kennels ……………………………………………………………………….

Section B

If transferred to other kennels Situated at: ….…………………………………… Date: ……………..……………………………….

**Dog Warden:** ……….……………………………

Section C

Outcome for Dog Reclaimed by Owner □

Rehomed □

Failed to rehome, then humanly euthanised □

Signed: …………………………………………………….

**Dog Warden / Poundkeeper**

Date: …………………………………...……………………

Humanly destroyed by: …………………………………………………………M.R.C.V.S.

Veterinary Surgeon Signature
