# Supplementary material for: A review of official data obtained from dog control records generated by the dog control service of county cork, Ireland during 2007
Source: Ir Vet J. 2012 Jun 8;65(1):10. doi: 10.1186/2046-0481-65-10 (PMC3489852; doi:10.1186/2046-0481-65-10)
Supplement: Additional File 2 — Owned dog voluntary surrender form completed by the dog warden when collecting a dog from its owner. [file 2046-0481-65-10-S2.doc]

**Additional File 2.** Owned dog voluntary surrender form completed by the dog warden when collecting a dog from its owner

**Cork County Council**

**Control of Dogs Act, 1986**

**SURRENDER FORM**

**Section A: (To be completed by owner)**

TO: Dog Warden..…………..……………………………………………………………………..

Address………………………………………………………………………………………………………………………………………………………………………………………………………

I,………………………………………, hereby declare that I am the owner of the dog described blow, and I am surrendering this dog to you, with regard to Section 12 of the Control of Dogs Act 1986.

**Dog Licence Number:** …………………………………………………………………….

(**Note:** For pedigree dogs, it is advisable that owners should be required to produce some form of identification)

**Description of Dog (Tick where appropriate)**

**Breed (if known)**…………………………………………..**Colour**………………..………..

Sex: Male □ Female □ Unknown □

Size: Large (20kg>) □ Medium (10-20kg) □ Small (10kg<) □

Personality: Friendly □ Nervous □ Timid □ Aggressive □ Destructive □ Barks aggressively □ Not to be trusted with children □

**SIGNATURE OF OWNER: ………………………………..………………………………**

**DATE: ………………………………**

**SIGNATURE OF DOG WARDEN: ………………………………………………………**

**DATE: ………………………………**

**Section B**

Transferred to other kennels situated at: ………… ….……………………………………

Signature: …………………………………….. Date: ……………………………….

**Section C**

Outcome for Dog Rehomed □

Failed to rehome, then humanly euthanised □

Signed: …………………………………………………….

**(Dog Warden / Poundkeeper)**

Date: …………………………………...……………………

Humanely destroyed by: ……………………………………………………….……M.R.C.V.S.

Veterinary Surgeon Signature
