# Supplementary material for: A review of official data obtained from dog control records generated by the dog control service of county cork, Ireland during 2007
Source: Ir Vet J. 2012 Jun 8;65(1):10. doi: 10.1186/2046-0481-65-10 (PMC3489852; doi:10.1186/2046-0481-65-10)
Supplement: Additional File 3 — List of the “Restricted breeds”. [file 2046-0481-65-10-S3.doc]

**Additional File 3.** List of the “Restricted breeds”

Control of Dogs (Restriction of certain dogs) Regulations 1991 and 1998 as amended, Section 4 subsection (1);

1. American Pit Bull Terrier
2. Bull Mastiff
3. Doberman Pinscher
4. English Bull Terrier
5. German Shepherd
6. Japanese Akita
7. Japanese Tosa
8. Rhodesian Ridgeback
9. Rottweiler
10. Staffordshire Bull Terrier

AND every crossbreed thereof
